# Appropriate Anti-Thrombotic/Anti-Thrombin Therapy for Thrombotic Lesions

**DOI:** 10.2174/157340312803217175

**Published:** 2012-08

**Authors:** Zafar Iqbal, Gurinder Rana, Marc Cohen

**Affiliations:** Division of Cardiology, Newark Beth Israel Medical Center, Newark, NJ

**Keywords:** Antithrombotic therapy, Coronary thrombus, Acute coronary syndrome.

## Abstract

Managing coronary thrombus is a challenging task and requires adequate knowledge of the various antithrombotic agents available. In this article, we will briefly analyze the risk-benefit profile of antithrombotic agents, with critical analysis of the scientific evidence available to support their use. Since thrombus consists of platelets and coagulation cofactors, an effective antithrombotic strategy involves using one anticoagulant with two or more antiplatelet agents. Unfractionated heparin traditionally has been the most commonly used anticoagulant but is fast being replaced by relatively newer agents like LMWH, direct thrombin inhibitors, and Factor Xa inhibitors.

In recent years, the antiplatelet landscape has changed significantly with the availability of more potent and rapidly acting agents, like prasugrel and ticagrelor. These agents have demonstrated a sizeable reduction in ischemic outcomes in patients with ACS, who are treated invasively or otherwise, with some concern for an increased bleeding risk. Glycoprotein IIb/IIIa inhibitors have an established role in high risk NSTE ACS patients pretreated with dual antiplatelets, but its role in STEMI patients, treated with invasive approach and dual antiplatelets, has not been supported consistently across the studies. Additionally, in recent years, its place as a directly injected therapy into coronaries has been looked into with mixed results. In conclusion, a well-tailored antithrombotic strategy requires taking into account each patient’s individual risk factors and clinical presentation, with an effort to strike balance between not only preventing ischemic outcomes but also reducing bleeding complications.

## INTRODUCTION

Treatment of thrombotic lesions in the coronary arteries, either in the setting of acute coronary syndrome (ACS) or new lesions formed during elective cases, represents a major challenge. Newly emerging and multiple available pharmacotherapies to address this potentially serious condition can add to this challenge. In this article we will assess the risk benefit profile of various antithrombotic agents, which can help in optimizing the antithrombotic strategy in the catheterization laboratory. 

Since the formation and propagation of thrombi involves interactions between activated platelets and the procoagulant factors of the coagulation cascade [1, 2], an optimal antithrombotic strategy consists of inhibiting both pathways enough to stop the development and propagation of thrombus, dissolve it in situ if possible, and balance this act against bleeding complications.

## ANTI-COAGULANTS 

[Please refer to Table **1** for the dosing of most commonly used anticoagulants]

### Heparins (UFH and LMWH)

UFH has been the most commonly used anticoagulant in the catheterization laboratory but its use is limited by variable dose response, narrow therapeutic index requiring frequent monitoring, and unpredictable effects despite using weight based nomograms [3-7].

Low-molecular weight heparins, on the other hand, have a more favorable profile with less plasma protein binding, no necessity for therapeutic monitoring, easier administration, and more consistent anti-coagulation as compared to UFH [8]. In the ESSENCE [9] and TIMI-11b [10], trials of UA/NSTEMI treated conservatively, LMWH had better efficacy outcomes compared to UFH.

In contrast, two other trials SYNERGY [11] and A-to-Z [12] did not show the superiority but did show non-inferiority for LMWH versus UFH in patients with NSTE ACS treated with early invasive strategy. There was higher incidence of TIMI major bleeding associated with LMWH in SYNERGY (9.1% vs 7.6%; p=0.008). However, it is important to note that in SYNERGY there were pre- and post-randomization treatment crossovers, and in patients treated consistently with one agent, there was a significant 18% relative risk reduction (13.3% vs 15.9%; HR 0.82, CI0.72-0.94) in favor of LMWH in the primary end point without any increase in bleeding [13]. Additionally, the trial protocol for the administration of intravenous enoxaparin was also violated in 9.2 % of patients. In a subsequent analysis, death and myocardial infarction occurred less frequently, though insignificantly, when the protocol was followed than otherwise (enoxaparin 12.3% vs UFH 14.4%; adjusted p = 0.25), with no difference in major bleeding. (3.0 vs 4.7%; adjusted p = 0.08) [14]. 

A subgroup analysis [15] of patients (n=4676) who underwent PCI in the EXTRACT TIMI 25 trial (LMWH vs. UFH in patients with STEMI treated initially with thrombolytics; n= 20,506) also showed that the primary combined end point of death and myocardial infarction at day 30 occurred less frequently in patients treated with enoxaparin versus UFH (10.7% vs 13.8%; p < 0.001), with similar rates of major bleeding (enoxaparin 1.4% vs UFH 1.6%; p=NS).

In a recent randomized trial, ATOLL (STEMI treated with primary angioplasty and intravenous Lovenox or unfractionated heparin; n=910), the primary end point consisting of death, complication of MI, procedure failure, and major bleeding at 30 days, occurred less frequently with the use of enoxaparin, without achieving statistical significance (28% vs 34%; RR 0.83, CI 0.68-1.01; p=0.063). The main secondary end point evaluating ischemic outcome (death, recurrent MI or ACS, or urgent revascularization) reached significance and demonstrated a 41% relative risk reduction in favor of enoxaparin (7% vs 11%; RR 0.59, CI 0.38-0.91; p=0.015). Bleeding incidence was equal between the two groups while net clinical benefit (death, complication of MI, or major bleeding) favored enoxaparin (10% vs 15%; RR 0.68, CI 0.48-0.97; p=0.030) [16].

**Fig. (1):**
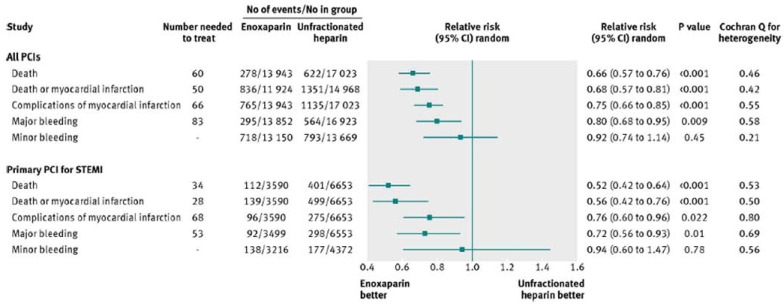
Pooled event rates and relative risk ratios for major end points in overall cohort of patients undergoing percutaneous coronary intervention (PCI) and in subgroup of patients undergoing primary percutaneous coronary intervention. STEMI=ST elevation myocardial infarction (printed with permission from BMJ, *BMJ 2012;344:e553 doi: 10.1136/bmj.e553)*

**Fig. (2):**
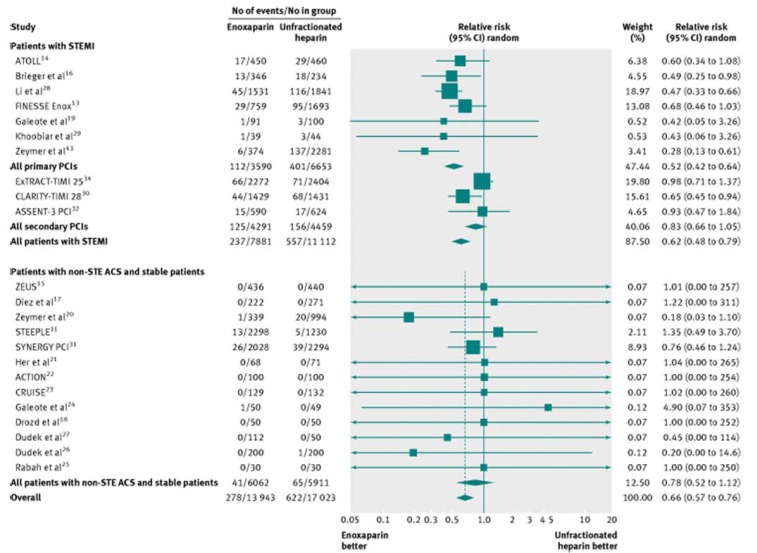
All cause mortality in patients undergoing percutaneous coronary intervention (PCI) treated with enoxaparin or unfractionated heparin. STEMI=ST elevation myocardial infarction; non-STE ACS=non-ST elevation acute coronary syndrome (printed with permission from BMJ, *BMJ 2012;344:e553 doi: 10.1136/bmj.e553)*

**Fig. (3):**
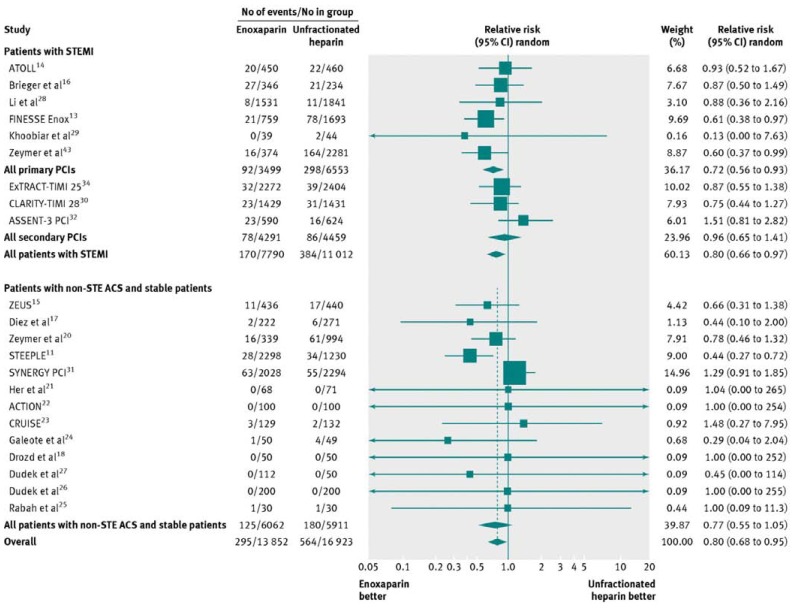
Major bleeding in patients undergoing percutaneous coronary intervention (PCI) treated with enoxaparin or unfractionated heparin. STEMI=ST elevation myocardial infarction; non-STE ACS=non-ST elevation acute coronary syndrome (printed with permission from BMJ, *BMJ 2012;344:e553 doi: 10.1136/bmj.e553)*

Johanne Silvain *et al*, performed a meta-analysis of 23 trials including 30,966 patients who underwent PCI (33.1% primary PCI for STEMI, 28.2% secondary PCI after fibrinolysis, and 38.7% with NSTE ACS or stable patients). The analysis showed that enoxaparin was associated with a 34% relative risk reduction (RR 0.66, 95% CI 0.58 to 0.77; P<0.001) and a 1.66% absolute risk reduction of mortality (NNT=60) [Fig. **1**, Fig. **2**], along with a significant reduction in major bleeding (RR 0.80, 95% CI 0.67- 0.95; P=0.009) [Fig. **3**]. Patients treated with primary PCI for STEMI had even more significant reduction in mortality (RR=0.52, CI 0.42 to 0.64; P<0.001) with a decrease in the incidence of major bleeding (0.72, 0.56 to 0.93; P=0.01) [17].

Overall, in light of the evidence stated above, LMWH (enoxaparin) appears to have a favorable risk benefit profile in comparison to UFH in patients who undergo PCI for ACS. 

### Direct Thrombin Inhibitors (DTI)

This class of anticoagulants inhibits thrombin directly as opposed to the indirect acting heparins and has a benefit with regard to no plasma protein binding and, hence, a more predictable response, along with improved inactivation of thrombin, both clot-bound and free [18]. The most commonly used DTI for treatment in ACS is Bivalirudin, a synthetic bivalent analog of hirudin. Two major trials have assessed Bivalirudin role in ACS using an invasive strategy, the ACUITY trial [19] and the HORIZONS-AMI trial [20]. 

In the ACUITY trial, 13,819 patients with NSTE ACS were enrolled, of which 7789 eventually underwent PCI. In the PCI group bivalirudin alone compared to UFH/LMWH with GPIIb-IIIa inhibitor (GPI) had similar ischemic outcomes (9%vs 8%, p=0·45), less major bleeding (4% vs 7%, p<0·0001, RR 0·52, 95% CI 0·40–0·66), and a trend in favor of better net clinical benefit (12% vs 13%, p=0·057; 0·87, 0·75–1·00) [21]. Although there is evidence that major bleeding in ACS is associated with higher mortality [22], a one year follow up of the ACUITY PCI subset did not show any difference in mortality or ischemic outcomes despite a reduction in major bleeding [23]. In a post hoc analysis of ACUITY, patients who received clopidogrel more than 30 minutes after PCI or not at all experienced higher ischemic events. In the setting of expected delay or inability to give clopidogrel, a “bivalrudin only” strategy may not be an advisable one [24].

In the HORIZON trial 3,602 patients presenting with STEMI treated with primary PCI were randomized to either a bivalirudin alone or an UFH/GPI arm. The Bivalirudin only arm had reduced 30-day net adverse clinical event rates (9.2% vs 12.1%; p=0.005) driven primarily by reduced bleeding with bivalirudin [4.9% vs 8.3%; p<.0001). At 1 year [25], and 3 years [26] the net adverse clinical event rates and major bleeding rates were reduced by 17% and 39%, respectively, yet major adverse cardiovascular events were still similar. Notably, bivalirudin use was associated with a significant increase in the rate of acute stent thrombosis (1.3% vs 0.3%; p=<0.001), though 30 day rates of stent thrombosis were not significantly different [20]. Additionally, 63.9% and 65.8% of patients in the bivalrudin “alone” arm of ACUITY and HORIZON respectively were pre-treated with open label UFH, which makes drawing definite conclusions difficult.

### Factor Xa Inhibitors

Factor Xa Inhibitors are a relatively newer class of anticoagulants which are rapidly expanding. Fondaparinux, has been studied in the OASIS-5 [27] and OASIS-6 trials [28] for NSTE ACS and STEMI. Although fondaparinux reduced bleeding events in these studies in comparison to heparins, its use in the patients who underwent PCI was associated with higher rates of catheter thrombosis and coronary complications, leading to hesitation in their use in patients progressing to PCI [28, 29]. Limited data from OASIS 5 and 6 demonstrated that the adjuvant use of UFH, in PCI patients treated with fondaparinux, reduced the incidence of catheter thrombosis to levels comparable to heparins. To understand the role of adjuvant UFH with fondaparinux, in the OASIS-8 trial, low dose UFH (50 U/kg) was compared with standard dose (60-85 U/kg) in 2026 patients, who presented with NSTE ACS and underwent PCI within 72 hours. Bleeding complications were similar with both doses while ischemic outcomes trended in favor of standard dose UFH (4.5% vs 2.9%; P=0.06). Catheter thrombosis rates were also very low (0.5% in the low-dose group and 0.1% in the standard-dose group, P=0.15) [30]. Therefore patients undergoing PCI, who are pre-treated with fondaparinux, should be administered standard dose UFH.

Otamixaban, an intravenous Xa inhibitor has been tested in two phase II trials; one in ACS (SEPIA-ACS) [31], and one in PCI (SEPIA-PCI) [32]. The phase III TAO trial is still underway to further evaluate the efficacy and safety of this agent (clinicaltrials.gov; NCT01076764).

## PLATELET INHIBITORS

Antiplatelet agents are required to inhibit platelet aggregation in the presence of activators such as ADP, thrombin, and collagen [33, 34], and thereby improving coronary blood flow. Please refer to (Table **1**) for the dosing of oral antiplatelets.

### Glycoprotein IIb/IIIa Inhibitors (GPI)

Since there are multiple pathways for platelet activation, current dual antiplatelet therapy (DAPT) is not enough in some cases to inhibit platelets effectively. GPI, by there inhibitory action on the final common pathway, can provide further platelet inhibition [35]. GPI have demonstrated reduction in the ischemic outcomes in ACS patients treated with an invasive strategy in multiple trials before the use of DAPT, but with a significant increase in bleeding [34-39]. Benefits of GPI were maintained in high-risk troponin positive patients pre-treated with clopidogrel in NSTE ACS patients who underwent PCI in the ISAR REACT 2 study [40].

In STEMI patients, treated with PCI and DAPT, the role of GPI has been conflicting [On-TIME 2 [41], FINESSE [42], BRAVE 3 [43], ADMIRAL [44]]. However, a meta-analysis of 10,085 STEMI patients treated with PCI demonstrated a mortality benefit with GPI in high-risk patients [45]. 

Coronary patency studies have also been conducted to demonstrate the efficiency of GPI, as patency has been shown to be a surrogate marker for 30 day mortality [46]. In the IMPACT-AMI trial, eptifibatide was given, in conjunction with fibrinolytic therapy to STEMI patients, and angiographic follow up at 90 minutes showed that the highest Eptifibatide dose achieved a 69% higher rate of TIMI grade 3 flow as compared to placebo (66% vs 39%, p=0.006), and an increased TIMI 2 and 3 flow in other eptifibatide groups [47]. Mixed results on angiographic patency rates and mortality are seen in other trials [48, 49]. An angiographic sub-study of CAPTURE in the post-PTCA angiograms demonstrated higher thrombus resolution rates with abciximab versus placebo (22% vs 43%; p=0.033) [50, 51]. In the PRISM-PLUS study, tirofiban and heparin versus heparin alone in UA/NSTEMI patients, reduced intracoronary thrombus burden (OR=0.77, p=0.022), improved perfusion grade, and decreased severity of the obstruction [52]. 

### Intracoronary (IC) Versus Intravenous (IV) GPI

The use of GPI as intracoronary agents has been tested on the basis of achieving higher local concentrations and, hence, better antiplatelet effects. In some small to moderate sized studies IC GPI has shown infarct size reduction, decrease in microvascular obstruction [53], improvement in the left ventricular function [54], and improvement in myocardial blush [55], but no significant difference in the clinical outcomes [56]. Interestingly, there have been meta-analyses in recent years [57, 58] which show a significant mortality benefit with IC GPI, although the studies included in these analyses are relatively small. Recently published, AIDA STEMI (n= 2065) [59] is the largest study which tested the role of IC GPI in STEMI patients undergoing primary PCI with hard clinical endpoints. The primary composite endpoint of all-cause mortality, recurrent infarction, or new congestive heart failure at 90 days did not differ with IC or IV use of GPI (7·0% vs 7·6%; OR 0·91; 95% CI 0·64-1·28; p=0·58). Importantly, lower event rates (8%) than expected (12%), coupled with relatively low risk patients (5 % Killip class 3 or 4, and left main or LAD was infarct related artery in 44 %), significantly reduced the power of the study. In summary the role of IC GPI still needs to be established.

The role of IC GPI was further studied in a recently published study (INFUSE AMI) [60], which consisted of 452 patients presenting with STEMI that involved proximal or mid-left anterior descending artery occlusion. Patients were randomized in a 2x2 factorial design to a single bolus of IC abciximab at the lesion site versus no abciximab, and manual aspiration thrombectomy versus no thrombectomy. Patients randomized to IC abciximab had a significant reduction in the primary end point of infarct size measured by cardiac MRI (15.2% vs 17.5 %; p=0.03), while thrombus aspiration, interestingly, had no significant impact on the outcomes with or without IC abciximab. 

### Aspirin and Adenosine Diphosphate (ADP) receptor blockers

The role of aspirin in ACS has been studied in multiple studies and two very large meta-analyses [61, 62] showing significant reduction in non-fatal MI and vascular death. Although the long term dose of aspirin is a much debated issue, the ACC/AHA guidelines recommend a loading dose of 162-325 mg of aspirin to all patients with ACS going for PCI.

**Fig. (4):**
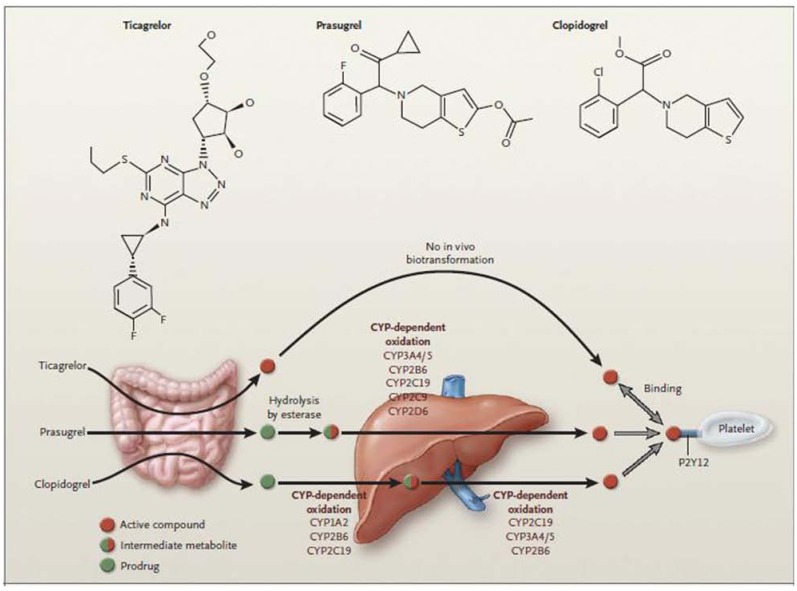
Ticagrelor, a cyclopentyl triazolopyrimidine, is rapidly absorbed in the intestine. The absorbed drug does not require further biotransformation for activation. It directly and reversibly binds to the platelet adenosine diphosphate (ADP) receptor P2Y12. The half-life of ticagrelor is 7–8 h. The thienopyridines prasugrel and clopidogrel are prodrugs. Their active metabolites irreversibly bind to P2Y12 for the platelet’s life span. After intestinal absorption of clopidogrel, it requires two cytochrome P-450 (CYP)-dependent oxidation steps to generate its active compound. After intestinal absorption of prasugrel it is rapidly hydrolyzed by means of esterases to an intermediate metabolite and requires one further CYP-dependent oxidation step to generate its active compound. Most of the CYP-dependent activation occurs in the liver. Relevant CYP isoenzymes involved in the activation of both clopidogrel and prasugrel are also shown. Their activity may be affected by genetic polymorphisms. (Adopted from NEJM 2009; 361:1108–1111).

Thienopyridines are antiplatelet agents directed against P2Y12 receptors on platelets (ADP receptors) and block a key pathway in the activation of the GPIIb/IIIa receptor [63]. Two are pro-drugs, clopidogrel and prasugrel, and require conversion to an active form in the gastrointestinal tract [64], while the other, ticagrelor, is the active agent [Fig. **4**].

Clopidogrel, a thienopyridine, demonstrated a reduction in death from cardiovascular causes, myocardial infarction, or stroke in comparison to aspirin alone in 12,562 patients with NSTE ACS (CURE trial) [65]. In a sub study of CURE [66] (patients undergoing PCI, n =2658), clopidogrel was associated with a 30% relative risk reduction compared to aspirin alone in CV death and myocardial infarction at 30 days (8.8% vs 12.6%, p=0.002) with no significant difference in major bleeding.

To identify an optimal loading dose of clopidogrel, in CURRENT OASIS-7 [67] 25,086 patients with ACS were randomized to either high dose clopidogrel (600 mg loading dose followed by 150 mg daily for one week then 75 mg daily) or standard dose clopidogrel (300 mg load followed by 75 mg daily), out of which 17,232 patients underwent PCI. Although the overall trial was neutral, the primary efficacy outcome (CV death, MI or stroke at 30 days), was reduced significantly in the subgroup who underwent PCI and received high dose clopidogrel, without an increased risk of major bleeding. This result should be interpreted with caution as it was a subgroup analysis. Similarly, high dose clopidogrel (600 mg) was associated with a lower incidence of ischemic events when compared to 300 mg in STEMI patients, who underwent PCI in the HORIZON AMI trial with an equal bleeding incidence [68].

Prasugrel is a thienopyridine with higher potency and a more rapid onset of action than clopidogrel [69, 70]. In TRITON-TIMI 38, 13,608 patients with ACS (10,074 NSTE ACS and 3,534 with STEMI) scheduled for PCI, were randomized to either prasugrel or clopidogrel. Ninety-nine percent of patients underwent PCI and 94% received at least one stent. The primary endpoint of death from CV causes, non-fatal myocardial infarction, or non-fatal stroke was significantly reduced in the prasugrel arm (9.9 % vs 12.1%, HR 0.81; 95%, CI 0.73-0.90; P<0.001),along with a reduction in stent thrombosis (2.4% vs. 1.1%; P<0.001) [71] (Table **2**). The prasugrel arm had a higher incidence of TIMI major bleeding (2.4% vs 1.8%, p=0.03) and demonstrated higher bleeding tendencies in patients with a prior stroke/TIA, age >75 years, or weight <60kg [72, 73].

Ticagrelor, a non thienopyridine oral P2Y12 receptor blocker [Fig. **4**], has been shown to have a favorable profile when compared to clopidogrel, secondary to reversible platelet inhibition, minimal hepatic activation, higher potency, and predictable platelet aggregation inhibition levels [74, 75]. In the PLATO trial, 18,624 patients presenting with ACS were randomized to standard treatment with either ticargrelor or clopidogrel [76]. At randomization, an invasive strategy was planned for 13,408 (72%) of the patients out of which 6,575 patients (49%) had presented with STEMI (Table **2**). The primary composite endpoint of cardiovascular death, myocardial infarction, and stroke occurred less frequently in the ticagrelor group than in the clopidogrel group (9·0% vs 10·7%, HR 0·84, 95% CI 0.75–0.94; p=0.0025), as well as all cause mortality (3.9% vs 5.0%; p=0.01) and stent thrombosis (1.3 % vs 2.0 %; p=0.0054), without an increase in major bleeding [11·6% vs 11·5%, 0·99 [0·89–1·10]; p=0·8803) [77].

Cangrelor, the first intravenous P2Y12 receptor blocker with very rapid onset of action and short half life [78, 79], failed to demonstrate any superiority over existing treatment strategies, in patients with ACS undergoing PCI [80, 81]. 

## DISCUSSION

Intracoronary thrombus encountered in the setting of ACS should be treated with at least two antiplatelet agents and one anticoagulant. If possible, all patients should receive aspirin with one ADP receptor blocking agent. When choosing ADP receptor blockers due consideration should be given to newer agents like prasugrel and ticagrelor, secondary to their more rapid onset of action, better efficacy profile, and improved ischemic outcomes in comparison to clopidogrel. This benefit must be judiciously weighed against a higher incidence of hemorrhagic complications associated with these agents. If for any reason oral antiplatelet agents cannot be administered in a timely fashion, intravenous GPI, with their rapid onset of action, may be considered as a reasonable alternative. Although use of GPI on top of DAPT is certainly recommended in high risk patients presenting with NSTE ACS with or without visible thrombus, evidence for their benefit in the STEMI population, assessed clinically or by surrogate endpoints, is inconsistent at best. Making definite recommendations about their role in STEMI patients, presenting with or without visible thrombus, is even more difficult in the absence of robust data, and their use perhaps should be reserved for high risk patients with large thrombus burden [82]. The impact of adjuvant GPI therapy on patients who underwent thrombectomy for intracoronary thrombus is also not adequately investigated. Thrombectomy in the TAPAS trial (thrombus aspiration compared with conventional treatment during primary PCI for STEMI), in which roughly 90 % of patients in both arms received intravenous GPI, was associated with better clinical and angiographic results [83]. Conversely, in the INFUSE AMI study [60] thrombectomy had no bearing upon the outcomes when used with or without intracoronary GPI. Two relatively large studies designed to assess the role of thrombectomy in patients with STEMI, TOTAL (ClinicalTrials.gov; Identifier: NCT01149044) and TASTE (ClinicalTrials.gov Identifier: NCT01093404) are underway and may shed some further light on this issue. Similarly, IC administration of GPI, although not supported by robust clinical data [59], has demonstrated improvement in the infarct size and may be used in patients with large visible thrombus. 

Regarding choice of anticoagulants, enoxaparin appears to have a better risk benefit profile in comparison to UFH, but lack of an antidote and increased bleeding with renal impairment should be kept in mind. When choosing bivalrudin as an anticoagulant, careful attention should be paid to the fact that, although bivalrudin is associated with reduction in bleeding complications and patients with higher bleeding risk might benefit from this strategy, cases in which GPI are used or expected to be used secondary to patient or lesion characteristics like heavy thrombus burden, bivalirudin may not provide additional benefit in terms of reduction in bleeding when compared to heparins. Additionally, a higher incidence of stent thrombosis in the initial phase and lack of an antidote should be considered. In patients pre-treated with fondaparinux intravenous UFH must be used during PCI. 

## CONCLUSION

Managing coronary thrombus entails individualization of therapy to each patient’s unique risk profile and depends on the setting in which coronary thrombus is encountered. An aggressive antithrombotic approach must always be tempered with keen attention to concomitant bleeding complications.

## Figures and Tables

**Table 1.** Dosings of Anticoagulants and Antiplatelet agents in the treatment of STEMI/NSTEMI/UA

	Patient Received Initial Medical Treatment (With an Anticoagulant and/or Fibrinolytic Therapy)	Patient Did Not Receive Initial Medical Treatent (With an Anticoagulant and/or Fibrinolytic Therapy)
ANTICOAGULANTS		
Bivalirudin [82,84]	Wait 30 minutes, then give 0.75 mg/kg bolus, then 1.75 mg/kg/hr infusion (*Class I rec)*	0.75 mg/kg bolus, then 1.75 mg/kg/hr infusion
UFH [82,84]	IV GPIIb/IIIa planned: target ACT 200-50 seconds No IV GP IIb/IIIA planned: target ACT 250-300 seconds HemoTec, 300-50 seconds Hemochron (*Class I)*	IV GP IIb/IIIa planned: 50-70 U/kg bolus to achieve an ACT of 200-50 seconds No IV GP IIb/IIIa planned: 70-100 U/kg bolus to achieve target ACT of 250-300 *(Class I)*
Enoxaparin [85-87]	With prior enoxaparin treatment, if last SC dose administered 8-12h earlier or if only 1 SC dose enoxaparin administered, an IV dose of 0.3mg/kg of enoxaparin should be given If last SC dose is administered within the prior 8h, then no additional enoxaparin should be given	0.5 mg/kg IV bolus
	If procedure is prolonged >2h, or if the operator needs stronger anticoagulation to manage peri-procedural complications, an additional IV bolus of enoxaparin (at ½ of original dose, 0.25 mg/kg) can be used	
Fondaparinux [84,88]	Because of the risk of catheter thrombosis, fondaparinux should not be used as the sole anticoagulant to support PCI 2.5 mg IV inititally for STEMI patients undergoing PCI 2.5mg SC with 50-60 U/kg IV bolus of UFH recommended	
THIENOPYRIDINES		
Clopidogrel [82,84]	If 600mg given orally, then no additional treatment A second loading dose of 300 mg may be given orally to supplement a prior loading dose of 300 mg *(Class I)*	Loading dose 300-600mg orally Maintenance dose of 75mg per day *(Class I)*
Prasugrel [85]	No data available to guide decisions	Loading dose 60mg orally Maintenance dose 10mg per day *(Class I)*
Aspirin [85]	Patients already taking daily aspirin therapy should take 81 mg to 325 mg before PCI (Class I)	Patients not on aspirin therapy should be given nonenteric aspirin 325 mg before PCI* (Class I)* It is reasonable to use aspirin 81 mg per day in preference to higher maintenance doses *(Class IIa)*
Ticagrelor [85]	No data available to guide decisions	Loading dose 180 mg orally Maintenance dose 90 mg twice daily *(Class I)*

**Table 2.** Trials Comparing Newer Antiplatelet Agents to Clopidogrel

Trial	Condition	Efficacy endpoint			Safety endpoint		
TRITON TIMI 38 (Prosugrel vs. Clopidogrel)	ACS patients scheduled for PCI (n=13608)	Death from cardiovascular causes, nonfatal myocardial infarction, or nonfatal stroke	9.9 vs. 12.1%	P<0.001	Major bleeding	2.4 vs. 1.8%	P=0.03
PLATO-Invasive (Ticagrelor vs. clopidogrel)	ACS patients scheduled for PCI (n=13408)	Death from vascular causes, myocardial infarction, or stroke	9.0 vs. 10.7%	P=0.0025	Major bleeding	3.2 vs 2.9%	P=0.37

## ABBREVIATIONS

UFH: = Unfractionated Heparin
LMWH: = Low Molecular Weight Heparin
ACS: = Acute Coronary Syndrome
STEMI: = ST Elevation Myocardial Infarction
NSTEMI: = Non ST Elevation Myocardial Infarction
NSTE ACS: = Non ST Elevation Acute Coronary Syndrome
UA: = Unstable Angina
PCI: = Percutaneous Coronary Intervention
NNT: = Number Needed to Treat
OR: = Odds Ratio
HR: = Hazard ratio
CI: = Confidence Interval
